# Identifying e-books authored by faculty: a method for scoping the digital collection and curating a list

**DOI:** 10.5195/jmla.2019.514

**Published:** 2019-01-01

**Authors:** Sonali Sugrim, Laura Schimming, Gali Halevi

**Affiliations:** E-Resources and Content Management Librarian, Gustave L. and Janet W. Levy Library, Icahn School of Medicine at Mount Sinai, New York, NY, 10029, sonali.sugrim@mssm.edu; Assistant Director, Gustave L. and Janet W. Levy Library, Icahn School of Medicine at Mount Sinai, New York, NY, 10029, laura.schimming@mssm.edu; Director, Gustave L. and Janet W. Levy Library, Icahn School of Medicine at Mount Sinai, New York, NY, 10029, gali.halevi@mssm.edu

## Abstract

Electronic books are a substantial component of many academic libraries. Many libraries aim to make their collections easily discoverable through curated lists. The authors’ library devised a methodology to identify and flag all e-books authored by our institution’s faculty using MARCEdit and Microsoft Access. We highlight some of the challenges in gathering a comprehensive list of titles, the process of formulating such a list, and the measures needed to actively curate e-books by faculty for both content already in the collection and newly published titles.

Many academic medical libraries seek to collect and curate materials that have been authored by their institutions’ faculty to serve their core library mission of promoting faculty scholarship. In recent years, the increased availability of large electronic books packages, coupled with unreliable or nonexistent author affiliation metadata and changing cataloging practices, have created a challenge for libraries to comprehensively identify books published by their own faculty authors. The authors describe a step-by-step solution used by librarians at the Icahn School of Medicine at Mount Sinai.

Our library serves a large academic medical institution, and the dean’s office requested that the library curate a list of books, both print and electronic, published by its authors. The purpose of the list was to showcase and provide access to publications dating back to 1968. We immediately recognized that this would be a challenging task for several reasons.

For many years, we had manually cataloged print books. We looked for affiliations on title pages and recorded them in the note field of the MARC record as well as labelled the print books with the given authors. As the collection expanded in size by thousands of e-books and transformed from print to mostly digital, it was no longer efficient or cost effective to manually scan each newly acquired title. Thus, large packages of e-books were regularly added to the collection without being methodically analyzed for possible faculty authors. Some publishers and book acquisition services successfully alerted librarians to individual books that had been authored by faculty; however, these services were not comprehensive across all publishers.

A scan of the literature revealed that the main cataloging and discoverability challenges that librarians have identified focused around using vendor supplied e-book records versus producing local cataloging records. The use of vendor supplied records has a set of challenges that relate both to e-book management and their discoverability. There are several examples:

- Vendors might not adhere to the MARC21 format [1].
- There are challenges in updating the catalog once records need to be updated or removed [2].
- There may be record quality gaps between vendors, and costs may be a challenge [3].
- The level of subject headings that is needed for appropriate discoverability might not be present [4].

Since both e-book availability and user demand for e-books are growing, libraries purchase large packages of titles at one time. These packages might include hundreds or even thousands of e-books. Managing these large e-book packages requires librarians to rely even more on vendor records for books. The general recommendations for catalogers have been to make good use of cataloging rules and keep up-to-date with current rules, make necessary changes to vendor-provided records, and add holdings to OCLC [3, 5].

While these guidelines are helpful to general knowledge on how to catalog, maintain, and make e-books available, the main challenge that we faced was the lack of affiliation metadata in these records. To identify Mount Sinai authors, we needed a method to search the collection of e-books by the affiliation of the authors. Unfortunately, we discovered that such a field did not normally exist in the e-books’ records. To address this challenge, we devised an innovative method to retrospectively search the collection and create an accurate list of faculty authored books.

## STEPS TO CREATE A CURATED LIST of LOCAL AUTHORS OF E-BOOKS

These steps outline the library’s process for identifying institutional authors in the collection using Microsoft Access, Microsoft Excel, MARCEdit, and OCLC’s WorldShare Management Service (WMS). Librarians using other discovery systems and software should be able to adapt the general instructions for use in their own environments.

1. Retrieve the MARC records for the library’s entire collection.
2. Gather a list of all faculty from the dean’s office.
3. Extract and compare the MARC records with the faculty list.
4. Flag MARC records, add a local holdings note, and create a Knowledge Bases and Related Tools (KBART) collection.
5. Add records to the curated list in WorldCat Local.

### Step 1: Retrieve collection data

Our library uses OCLC’s WMS for all e-resources and cataloging management. To retrieve the current collection’s MARC records, we selected the option to create a collection using WMS’s Metadata tab and then selected “query collection” for the collection type. We selected a name for the collection and used the WorldCat selection criteria, *li* for library and colon OCLC symbol (i.e., li: OCLC symbol). OCLC exports 100,000 records per file, so large library collections may take a few days to retrieve and may be exported in multiple files or lists.

**Figure 1:**
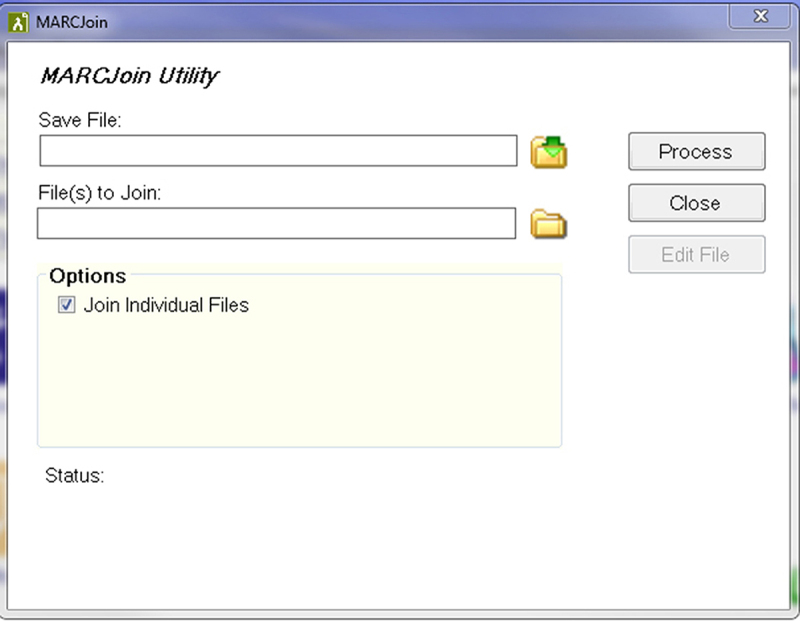
MARCJoin utility

We used MARCEdit’s MARCJoin utility to combine the multiple lists of MARC records into one list (Figure 1). In MARCEdit, the Export Tab feature allowed us to create an Excel file with fields of our choosing from the MARC record.

The basis for comparing MARC records with the faculty list was the author’s name, so the 100 and 700 fields are crucial. We chose the OCLC Number (001), ISBN (020), Statements of Responsibility (245), Publication Year (260), and/or Copyright Year (264) in addition to the Authors’ name fields (100 and 700) to export into the Excel file. These fields helped to identify records and flag duplicates, date ranges, and editions.

After we extracted an Excel list, we reviewed the list for duplicates and noticeable errors. Errors included unusual characters that did not always populate correctly. Also, it was important to include authors’ full names, including middle names or initials, in order to avoid adding a title with an author who was not affiliated with the institution. We then imported the revised list into Microsoft Access and labeled it accordingly.

### Step 2: Gather a list of institutional faculty

The dean’s office provided a list of current faculty members. We edited the list to remove duplicate names and uploaded and labelled the file in Microsoft Access.

### Step 3: Extract and compare the MARC records with the faculty list

The “External Data” tab in Microsoft Access allows Excel files to be imported. The next step was to select all the fields in the MARC records list and establish a relationship with the authors of both lists.

After both files were imported into Microsoft Access, we used the “Create Tab” menu and the Query Design icon to design the query to compare the MARC records list and faculty list. Fields included ISBN, OCLC Number, Author, and Title. Note these are all customizable and may vary due to a librarian’s preferences.

**Figure 2:**
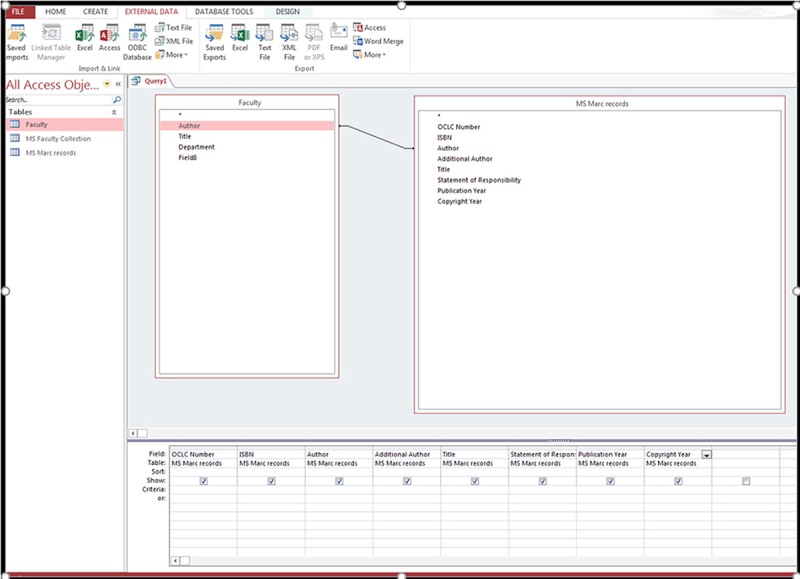
Microsoft Access query

Once these fields were populated, we established a relationship or link between the lists by matching the authors’ names from both files. This was done by selecting the authors’ field on the MARC records list and dragging it to the Author field on the faculty list, as shown in Figure 2.

Then we created and labelled a table to import the authors’ collection. Next, we selected the run icon to run the query. Since we selected both the 100 and 700 fields from the MARC record list, we ran the 100 field first and then the 700 field using the append feature. That is, we had 2 author fields in the MARC records list called Authors 1 (100) and Authors 2 (700). We joined Authors 1 (100) with the authors from the faculty list and ran the query; then, we did the same for the Authors 2 (700) field. Once the match was completed, we checked for duplicates and then exported the data as an Excel file. The data can be exported and duplicates can be removed in Excel, depending on the librarian’s preference.

### Step 4: Flag MARC records and create a KBART collection

After retrieving the faculty authors list, we thoroughly reviewed the data to remove records in which authors might have the same names but not be associated with the institution. It was fairly simple to spot e-book records that were not medical or were out of scope and thus likely by a different author. These errors were few and could be eliminated if the full names including middle initials or names were used in the query as the final list became more accurate. This list focused on main authors; however, the same process can be used to identify contributing authors.

Once the records were assessed, we contacted OCLC to request a data sync to add a local Mount Sinai author note to the flagged MARC records. In cases where the faculty authors list was small, a note could be added manually in the OCLC’s Local Holdings Record (LHR). We chose to add “Mount Sinai Author” in the public note ($z) of the 876 field of the local holdings record. There is some flexibility as to where a note can be added in the MARC records so librarians have some choice in the matter. In addition, we created a KBART collection with our current authors’ titles.

### Step 5: Add records to a curated list in WORLDCAT local

**Figure 3:**
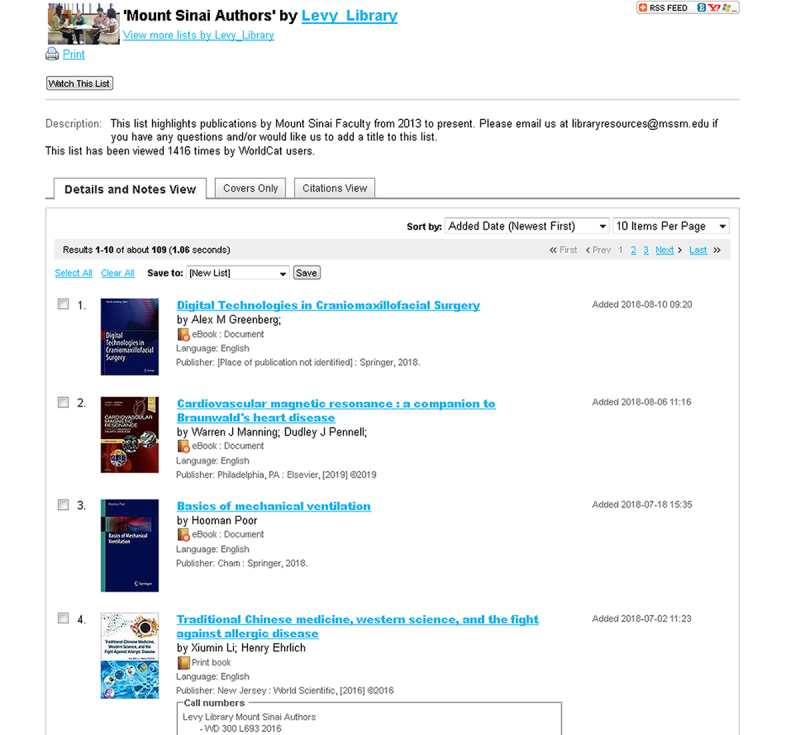
WorldCat local curated list

Finally, the completed list of affiliated e-books was made available on the library’s website using WorldCat Local’s List feature (Figure 3). This curated, easy-to-update list offered a seamless method for sharing the list with the dean’s office and other interested groups and for publicizing Mount Sinai research and publications.

## DISCUSSION

We plan to perform this query every three months in order to flag new authors and newly added e-books. This process does not help for newly published titles that are not currently in the library’s collection. This method establishes a curated authors list and a method for periodically assessing the collection to ensure that all associated authors’ publications are flagged, properly managed, and made accessible to users. It is advisable to note that many publishers do not flag affiliated authors. E-book acquisition services such as GOBI do flag affiliated authors. Sales representatives are able to guide librarians on how to set up alerts and create custom queries in order to identify newly published content by institutional authors.

## References

[1] Wilkins V (2007). Managing e-books at the University of Derby: a case study. Program.

[2] Mincic-Obradovic K (2009). Ten years on: e-books at the University of Auckland Library. Serials.

[3] Martin KE, Mundle K (2011). Cataloging e-books and vendor records. Libr Resour Tech Serv.

[4] Rossmann D, Foster A, Babbitt E (2009). E-book MARC records: do they make the mark?. Serials.

[5] Dinkelman A, Stacy-Bates K (2007). Accessing e-books through academic library websites. Coll Res Libr.

